# Supplementary material for: An open-source framework for large-scale, flexible evaluation of biomedical text mining systems
Source: J Biomed Discov Collab. 2008 Jan 29;3:1. doi: 10.1186/1747-5333-3-1 (PMC2276192; doi:10.1186/1747-5333-3-1)
Supplement: Additional file 1 — Evaluation platform source code. The additional file contains the software evaluation framework discussed in this paper. The most current release can be downloaded from SourceForge.net [1]. See the accompanying README file for instructions on installation and use. Also included in the distribution is a collection of UIMA wrappers for some commonly used BioNLP tools and annotated corpora. [file 1747-5333-3-1-S1.zip › bionlp_uima_repository-1.0.1/build/test/reports/unit/edu/uchsc/ccp/util/nlp/mention/SlotMentionTest.html]

Unit Test Results: edu.uchsc.ccp.util.nlp.mention.SlotMentionTest


# Unit Test Results

|  |  |
| --- | --- |
|  | Designed for use with JUnit and Ant. |

---

### Class edu.uchsc.ccp.util.nlp.mention.SlotMentionTest

| Name | Tests | Errors | Failures | Time(s) |
| --- | --- | --- | --- | --- |
| SlotMentionTest | 6 | 0 | 0 | 0.014 |

## Tests

| Name | Status | Type | Time(s) |
| --- | --- | --- | --- |
| testEquals | Success |  | 0.008 |
| testConstructor | Success |  | 0.000 |
| testGetSingleLineRepresentation | Success |  | 0.001 |
| testAddSlotValue | Success |  | 0.000 |
| testGetFirstSlotValue | Success |  | 0.000 |
| testSetFirstSlotValue | Success |  | 0.000 |

Properties »
